# Fucoidan-Derived Functional Oligosaccharides: Recent Developments, Preparation, and Potential Applications

**DOI:** 10.3390/foods12040878

**Published:** 2023-02-18

**Authors:** Min Wang, Suresh Veeraperumal, Saiyi Zhong, Kit-Leong Cheong

**Affiliations:** 1 Guangdong Provincial Key Laboratory of Aquatic Product Processing and Safety, Guangdong Province Engineering Laboratory for Marine Biological Products, Guangdong Provincial Engineering Technology Research Center of Seafood, Guangdong Provincial Science and Technology Innovation Center for Subtropical Fruit and Vegetable Processing, College of Food Science and Technology, Guangdong Ocean University, Zhanjiang 524088, China; 2 Postgraduate College, Guangdong Ocean University, Zhanjiang 524088, China; 3 Department of Biology, Shantou University, Shantou 515063, China

**Keywords:** fuco-oligosaccharides, brown algae, preparation, biological activities, prebiotics

## Abstract

Oligosaccharides derived from natural resources are attracting increasing attention as both food and nutraceutical products because of their beneficial health effects and lack of toxicity. During the past few decades, many studies have focused on the potential health benefits of fucoidan. Recently, new interest has emerged in fucoidan, partially hydrolysed into fuco-oligosaccharides (FOSs) or low-molecular weight fucoidan, owing to their superior solubility and biological activities compared with fucoidan. There is considerable interest in their development for use in the functional food, cosmetic, and pharmaceutical industries. Therefore, this review summarises and discusses the preparation of FOSs from fucoidan using mild acid hydrolysis, enzymatic depolymerisation, and radical degradation methods, and discusses the advantages and disadvantages of hydrolysis methods. Several purification steps performed to obtain FOSs (according to the latest reports) are also reviewed. Moreover, the biological activities of FOS that are beneficial to human health are summarised based on evidence from in vitro and in vivo studies, and the possible mechanisms for the prevention or treatment of various diseases are discussed.

## 1. Introduction

**Figure 1:**
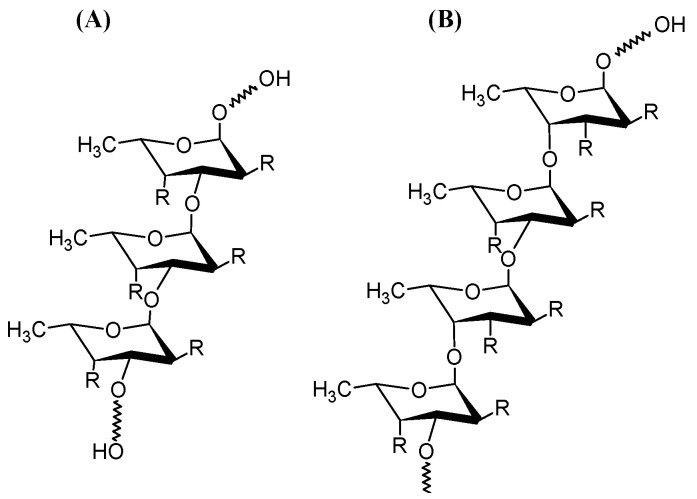
The chemical structure of fucoidan. (**A**) backbone of α-(1→3)-L-fucopyranose residues; (**B**) backbone alternating α-(1→3)-linked and α-(1→4)-linked L-fucopyranose residues. R = OH or OSO_3_^−^.

Fucoidan is a polysaccharide mainly composed of fucose, with a high sulphate content (more than 20%). This carbohydrate moiety is found in marine resources, generally in brown algae, sea cucumbers, and sea urchin eggs (as indicated in Table 1). The chemical structure of fucoidans consists of a backbone of α-(1→3)-L-fucopyranose residues, or alternating α-(1→3)-linked and α-(1→4)-linked L-fucopyranose or α-(1→2)-L-fucopyranose residues (Figure 1), with different substitutions, such as fucose, sulphate, acetate, and uronic acid [1,2]. Fucoidan presents a wide range of pharmaceutical activities, including antioxidant, anticancer, immunomodulatory, anticoagulant, and antimicrobial activities [3,4].

In fact, the preparation and structural characterisation of fucoidan seem to be well elucidated. However, haemorrhagic risk, high molecular weight (MW), high viscosity of fucoidan, and a poor dissolution rate in solution may limit their successful application in functional foods and pharmaceuticals [9,10]. Fucoidans often differ in terms of structural diversity and complexity; therefore, standardisation and quality assurance of fucoidans are challenging. Therefore, there has been a recent increase in interest in degrading fucoidan into fuco-oligosaccharides (FOSs) or low-MW fucoidan (LMWF) to broaden potential applications.

Oligosaccharides consist of a mixture of 2–20 monosaccharides, with a variable extent of polymerisation. They have attracted increasing interest owing to their nutritional and biological properties [11]. Many functional oligosaccharides, such as fructo-oligosaccharides, galacto-oligosaccharides, xylo-oligosaccharides, manno-oligosaccharides, and isomalto-oligosaccharides, have been widely used as prebiotics in food application [12,13,14]. Commercial prebiotics are derived from terrestrial plant oligosaccharides. However, research is underway to explore new prebiotics from natural resources, especially from renewable sources. Marine algae have unparalleled advantages as easily renewable and extremely abundant resources, as they grow in seawater and avoid the demand for freshwater. FOSs, also called LMWF, is an oligomer of fucoidan, which is normally obtained from brown algae. FOSs have a degree of polymerisation (DP) < 20 and an average MW of less than 10 kDa. FOSs also show a prebiotic ability and can be fermented by the gut microbiota, exert a range of physiological effects on body functions and improve human health [15]. Therefore, FOSs and other marine algae oligosaccharides are considered promising prebiotics.

This review provides a comprehensive background to FOSs, and evidence of their functional effects on human health. To this end, a detailed review of the current state of research on the preparation, purification, and characterisation of FOSs is presented. Thereafter, the potential health benefits of FOSs are discussed explicitly.

## 2. Preparation of Fucoidan and/or Raw Materials for Fuco-Oligosaccharide Production

**Figure 2:**
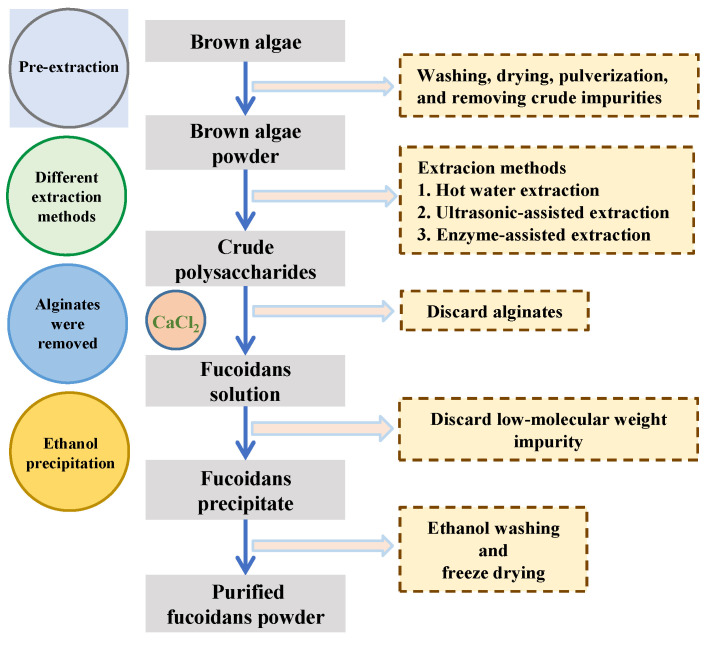
Schematic diagram of the extraction and preparation of fucoidan from brown algae.

The advantages of FOSs include high water solubility, low viscosity, and good biological activity. However, FOSs are seldom found in natural resources. Therefore, the degradation of fucodain is necessary for the production of FOSs. Figure 2 shows a singular diagram of the process of preparing fucoidan from brown algae. The extraction processes are based on aqueous (water, acidic, or alkaline solution) extraction; the polysaccharides are precipitated by ethanol; proteins are removed by the Sevag or enzymatic method, and the low-molecular-weight impurity is discarded by diafiltration [16]. The Sevag method is widely used to discard protein. It was first exploited by Sevag et al., as they used an organic solvent, including chloroform and n-butanol, to precipitate protein from sample solution [17]. An acid or calcium chloride solution added to the polysaccharide mixture has been commonly used to separate alginic acid from fucoidan. Béress et al. precipitated the alginic acid by adding glacial acetic acid, and the process was completed after 12 h at 4 °C [18]. Furthermore, column chromatography, such as ion-exchange and size-exclusion methods, can be used to obtain fucoidan [19]. Currently, three major processes exist to obtain FOSs from fucoidan: degradation by mild acids, enzymes, and radical methods. The mild acids and radical degradation methods are considered to be random and non-specific in degrading fucosidic linkages, while enzymatic method recognizes the specific substrate to cleave fucosidic linkages.

## 3. Preparation of Fuco-Oligosaccharides

### 3.1. Mild Acid Hydrolysis

Mild acid hydrolysis usually uses dilute acid solutions, such as sulphuric acid, hydrochloric acid, and trifluoroacetic acid, at suitable heating temperatures and times. FOSs mixtures are obtained after hydrolysis and subsequent neutralisation. Mild acid hydrolysis seems to offer a simple, low-cost production, and an easy operation, especially to fulfil requirements in the laboratory. Therefore, the mild acid hydrolysis method is always adopted by laboratories to screen for potential biological activities of FOSs. An aliquot of 0.05 mol/L sulphuric acid solution was used to degrade *Nemacystus decipiens* fucoidan at 80 °C for 2 h to obtain FOSs with a DP ranging from 2 to 9, and the FOSs showed antithrombotic activity [20]. A high-MW fucoidan, derived from *S*. *hemiphyllum*, was degraded by 0.01 mol/L hydrochloric acid at 85 °C for 15 min to obtain FOSs with a MW of 800 Da. The result showed skin-protective effects against ultraviolet B damage [21].

However, the degradation method is non-specific and may produce a series of FOSs, which increases the challenge of purifying FOSs. Moreover, the sulphated group in fucoidan is acid-labile and easily hydrolysed during acid hydrolysis. Mild acid hydrolysis (parameters used 0.005–0.01 mol/L trifluoroacetic acid at 60–100 °C for 2–4 h) was conducted on fucan sulphate derived from *S*. *herrmanni*, to obtain a number of FOSs. They contained two series of FOSs: 2-desulphated and 2-sulphated residues at the reducing ends of FOSs [22]. Pomin et al. found that in the dilute acid hydrolysis of fucoidan, the 2-sulphate ester group is initially cleaved, subsequently degrading the glycosidic bonds between the non-sulphated residue and the 4-sulphate group [23]. The kinetic constant of mild acid hydrolysis on sulphated fucan derived from three sea cucumber species, *I*. *badionotus*, *H*. *floridana*, and *L*. *variegatus*, with core α (1→3)-linked fucose, degraded the sulphate group substitution at the 2-O and 4-O-positions. The mild acid hydrolysis progress was under 0.05 mol/L sulphuric acid at 60 °C for 9 h, and selective 2-desulphation in sulphated fucan and tetrasaccharides FOSs were obtained [24].

Mild acid hydrolysis is suitable for a large-scale production of FOSs. Future studies on the kinetics and mechanism of mild acid hydrolysis should focus on providing fundamental data for industrial application in the large-scale production of FOSs.

### 3.2. Enzymatic Hydrolysis

Several hydrolytic enzymes that catalyse the hydrolysis of polysaccharides have been characterised. Normally, the breakdown of fucoidans involves the degradation of glycosidic linkages by fucoidanases (EC 3.2.1.44) and α-L-fucosidases (EC 3.2.1.51, EC 3.2.1.63, EC 3.2.1.111, and EC 3.2.1.127), which converts fucoidans into FOSs [25]. The enzymes that produce FOSs are produced by marine microorganisms and include endo- and exo-enzymes [26]. The well characterized endo-enzymes for fucoidan hydrolysis include endo-fucanases FcnA, FFA1, FFA2, Fp273, Fp277, Fp279, FWf1, FWf2, Fhf1, Fhf2 of the GH107 family and endo-fucanase FunA of the GH168 family, while the α-L-fucosidase is exo-enzymes and described for the families of glycoside hydrolases (GH) 29, 95, and 141 [25,27,28]. Enzymatic depolymerisation is highly selective for specific substrates and efficient for producing the desired end-product under selective method conditions. Therefore, with the enzymatic method, unlike the mild acid hydrolysis method, it is possible to achieve hydrolysate of crude fucoidan yielding FOSs.

The extent of enzymatic action is strongly dependent on operating conditions, including temperature and pH. Fucoidan with a MW of approximately 400 kDa from *F*. *distichus* was incubated together with endo-α-(1,4)-fucoidanase from the marine bacterium *Formosa haliotis* (Fhf1Δ470), in Tris-HCl buffer (pH 8) at 37 °C for 1 d, yielding FOSs with a MW of 2 kDa [29]. The study demonstrated the use of the enzymatic technique to screen out the tailored fucoidan functional group (FOSs) to specifically improve bone regeneration [29]. Enzymatic depolymerisation of *L*. *japonica* fucoidan by fucoidanase—which is produced by *Flavobacteriaceae* RC2-3—yielded FOSs with a MW < 2 kDa (96.3%) after incubation at 37 °C for 10 h. The FOSs products showed an excellent radical scavenging activity and inhibition of tyrosinase activity, rendering them suitable for application as cosmetic skin-whitening additives [30]. Another advantage of the enzymatic method is that the sulphated groups in FOSs may be preserved during the depolymerisation process. A fucoidanase obtained from the digestive glands of mollusc *Lambis* sp., specifically, degraded (1→3) and (1→4)-α-L-fucans derived from *F*. *evanescens* and *F*. *vesiculosus*, yielding sulphated FOSs, without any effect on the sulphate group [31].

To overcome problems of using the enzyme, including those owing to its high cost and poor stability, the immobilised enzyme technique is preferred. The immobilised enzyme technique has the advantages of maintaining enzymatic activity, ease of recovery after coating monolithic substrates, and easy scaling up of processes for industrial application [32].

The enzymatic method is considered a good choice for producing FOSs for applications in the food, cosmetic, and pharmaceutical industries. Therefore, screening for new enzymes that can degrade fucoidan has become an increasingly important focus of scientists and industrial engineers; in particular, searching for new endo-fucoidanases and their subsequent kinetic analysis, molecular cloning, and immobilisation techniques to provide valuable fundamental knowledge to produce highly active FOSs.

### 3.3. Radical Depolymerisation

Radical depolymerisation is a process that uses radicals to attack glyosidic linkages of polysaccharides to form oligosaccharides. The radicals, including HOO^−^•, •O_2_^−^, and HO•, can be generated by hydrogen peroxide (H_2_O_2_) and a catalytic metal system. Depolymerisation by H_2_O_2_ involves radicals (HOO•, •O_2_^−^, and HO•) attacking the glycosidic linkages [33]. This method has the advantages of good reproducibility and controllability. The main parameters that influence the efficacy of depolymerisation include the temperature, concentration of H_2_O_2_, reaction time, and substrate concentration.

Radical depolymerisation of fucan sulphate from *S*. *herrmanni* was performed by adding H_2_O_2_ and copper (II) acetate monohydrate. On average, the MW of fucan sulfate was degraded from 790.8 kDa to 8.32–22.11 kDa. The resulting products exhibited good anticoagulant activity [34]. Moreover, a combination of ascorbic acid and H_2_O_2_ was used to degrade fucoidan at 25 °C for 16 h, to obtain a fraction with the MW of approximately 3 kDa, which exhibited anti-lung-cancer activity in vitro [35]. Qi et al. reported that radical depolymerisation can maintain the sulphate content or other functional units in the degradation process [36]. Qi et al. degraded *U*. *pinnatifida* fucoidan using a photocatalytic method, which processes the reaction in H_2_O_2_ and TiO_2_ under xenon light for 3 h. While the MW of 190 kDa was degraded to 3 kDa, the resulting product exhibited anticoagulant activity [36].

Moreover, photoirradiation techniques for the degradation of polysaccharides have been attracting increasing attention. These techniques provide efficient degradation methods for the production of FOSs from fucoidan, because photoirradiation can be achieved at room temperature and under normal pressure. Ultraviolet and gamma radiation are typically used to degrade fucoidan. They are an energetic form of electromagnetic radiation, with a short wavelength and high energy. Water radiolysis is the major effect of gamma irradiation of solutions. Higher gamma irradiation doses may result in a lower MW of fucoidan: the MW of *F*. *vesiculosus* fucoidan degraded from 210 kDa to 85 kDa and 7 kDa following irradiation doses of 8 kGy and 100 kGy, respectively, while the latter degraded product showed the highest antioxidant activity [37]. In addition, photoirradiation combined with radical depolymerisation increased the efficiency of FOSs production. The average MW of fucoidan decreased to 6.7 kDa at an irradiation dose of 20 kGy, while the degradation efficiency increased by 5% when a 10% H_2_O_2_ concentration was used in combination with gamma radiation. The hydroxyl radical scavenging abilities of degraded product of FOSs were more effective than fucoidan [38]. Park and Choi also used a combination of gamma radiation at a dose of 100 kGy and H_2_O_2_ to degrade *U*. *pinnatifida* fucoidan from MW 378 kDa to 6 kDa. Park and Choi found that degraded fucoidan has a higher inhibitory activity against tyrosinase and antioxidant activity than fucoidan [39].

## 4. Purification of Fuco-Oligosaccharides

**Figure 3:**
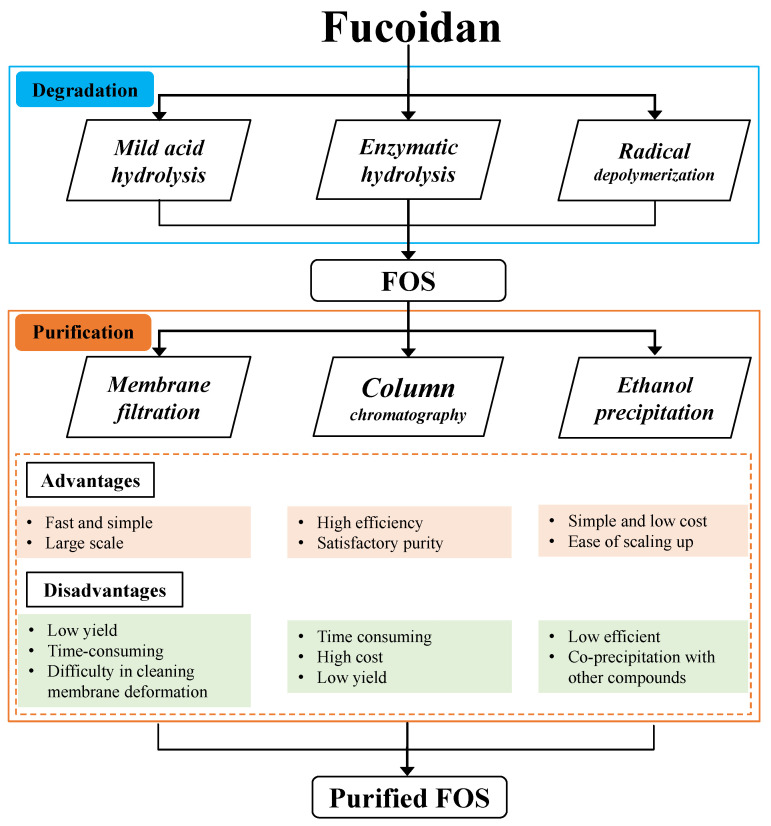
Schematic diagram of FOS degradation and purification processes.

The mixtures of FOSs produced by degradation methods include a variety of compounds, i.e., monosaccharides, FOSs ranges of different DPs, and large MW fucoidan. To obtain high-purity FOSs (for use in studies of the structure–function relationship and mechanism, and for applications in functional food or pharmaceutical areas), some suitable purification steps should be performed. Figure 3 shows a schematic diagram of the production and purification of FOSs.

Step-gradient ethanol precipitation is a convenient and time-saving method for the large-scale production of FOSs. Different MWs can be prepared by adding an ethanol gradient at a final concentration of 10–80%. However, this method yields a broad MW distribution of FOSs, and it is difficult to obtain high-purity FOSs. For example, Fernando et al. performed a step-gradient ethanol precipitation to exclude high-MW fucoidan, and a broad MW distribution of FOSs, ranging from 8 to 25 kDa, was achieved [40]. Thus, ethanol precipitation is useful as a preliminary step for discarding high-MW fucoidans or proteins.

Membrane filtration is another green technique for the purification of FOSs, and its performances are quite similar to gradient ethanol precipitation, which obtained a broad MW distribution of FOSs. Zhao et al. purified a degraded fucoidan mixture, using an ultrafiltration membrane (10 kDa and 2.5 kDa MW cut-off) to obtain the FOSs fraction, and the fraction range 2.5–10.0 kDa was collected [9]. The degraded fucoidan samples passed through a 30-kDa-MW cut-off membrane and subsequently through a 1-kDa membrane, obtaining an FOSs fraction MW range of 1–30 kDa [41]. Dialysis bags with different MW cut-off membranes have been used to obtain MW values of lower than 10 kDa fraction [42].

Column chromatography, including ion-exchange chromatography and size-exclusion chromatography, is widely used for fractionation oligosaccharides during purification. Size-exclusion chromatography has been used to fractionate oligosaccharides according to their MW, and this is suitable for the purification of FOSs and achieving high purity. Commercially available size-exclusion chromatography resins have been used to purify FOSs. The purified FOSs fractions are suitable for structural identification and pharmaceutical activity investigation. After mild acid hydrolysis the fucoidan hydrolysate sample was purified using a Bio-Gel P-2 column, and a high-purity fucotrioligosaccharide was obtained [43]. A mixture of oligosaccharides, produced from acid hydrolysis, was further purified using Bio-Gel P-4 and P-2, to obtain a high-purity yield of 6–9% of monosulphated fucobiose and monosulphated fucotriose [44]. Ion-exchange chromatography for FOSs purification proceeds according to the sulphate content of the FOSs, which leads to different ionic interactions with the resins. The FOSs mixture produced from fucoidan was degraded by the enzymatic method and further purified by ion-exchange chromatography on Q-Sepharose, to yield DP 4–10 FOSs with different sulphate contents [45].

## 5. Biological Activities of Fuco-Oligosaccharides

### 5.1. Antioxidant Activity

Numerous studies have reported the importance of antioxidants in human health systems. Antioxidants counteract oxidative damage to nucleic acids, lipids, peptides, and proteins that causes diseases including cancers, chronic inflammation, cardiovascular diseases, neurodegenerative disorders, and metabolic diseases [46,47]. The search for new and safe antioxidant components obtainable from natural sources, and the investigation of their antioxidant properties has attracted substantial interest worldwide.

The in vitro antioxidant activity of components of interest are normally tested in more than one assay model, as different antioxidant methods vary and are established based on their various scavenging mechanisms [48]. Previous studies have shown that antioxidant activities are affected by the MW of oligosaccharides, and several reports have demonstrated that antioxidant activities increase with a decrease in the MW of oligosaccharides [49]. Hou et al. compared different LWMF, which were degraded from *Saccharina japonica* fucoidans; they revealed that the hydroxyl scavenging ability was the highest in 1.5–4.0 kDa and 80 kDa fractions, while the reducing power increased as the MW decreased below 13 kDa [50].

The antioxidant activity of FOSs is also influenced by its degree of polymerisation and sulphate content. Different degrees of polymerisation (DP2, DP4, and DP6) of oligosaccharides were obtained from *S*. *thunbergii* fucoidan by acid degradation. The DP 2 oligosaccharides had lower hydroxyl radical scavenging activity, 1,1-diphenyl-2-picrylhydrazyl radical scavenging and reducing power than DP 4 and DP 6. The DP2–6 oligosaccharides had a lower reducing power when compared with sulphated oligosaccharides [51].

On the contrary, FOSs with antioxidant activities also play important roles in the antioxidant defence system in vivo. FOSs act as a frontline defence by suppressing the formation of potentially harmful metabolic by-products (reactive oxygen and nitrogen species) that can have deleterious effects on the body and cause damage to host biological structures [52]. A crude LWMF, obtained from *S*. *autumnale* by protamex-assisted hydrolysis, showed significant prevention against H_2_O_2_-induced oxidative stress in a zebrafish model, and demonstrated that it improved the heart rate, decreased the generation of ROS, and suppressed lipid peroxidation in zebrafish embryos [53]. Notably, FOSs have also been demonstrated to improve the skin antioxidant network. The UVB-irradiated skin-damaged mouse models showed an increase in glutathione and decreased myeloperoxidase, malondialdehyde, and superoxide after treatment with FOSs. FOSs treatment suppresses the progression to oxidative stress and improves innate antioxidant-related enzyme activities [54].

In short, FOSs have promising potential applications as naturally derived antioxidant or radical scavenging agents in the pharmaceutical, nutraceutical, cosmetic, and food industries.

### 5.2. Anticancer Activity

Cancer is a major health problem and the second leading cause of death worldwide. More than half of all cancer cases and tumour development processes in the body are considered preventable [55]. As anticancer drugs have adverse side effects on several tissues, including the brain, heart, and gastrointestinal tract, doctors and biologists have been interested in the application of compounds derived from natural products to prevent cancer development in the body. Numerous studies have demonstrated a relationship between anticancer activities and highly functional compounds derived from natural resources [56,57]. Marine algae are among the most abundant resources and remain under-exploited. They contain a variety of nutrients, among which, marine algae polysaccharides and their derived oligosaccharides have attracted considerable interest. In our previous review, we summarised a variety of direct and indirect anticancer activities of marine algal polysaccharides that inhibit or affect cancer and tumour cells [58]. Marine algae polysaccharides exhibit anticancer activities with different reported signalling pathways, including mitogen-activated protein kinase, nuclear factor-kappa B, phosphoinositide 3-kinase/protein kinase B, and Wnt/β-catenin signalling pathways, which have been summarised and discussed in a previous review [58]. The increasing recognition of bioactive oligosaccharides has accelerated research into their potential anticancer activity. We believe that FOSs may have promising potential applications in cancer therapy.

**Figure 4:**
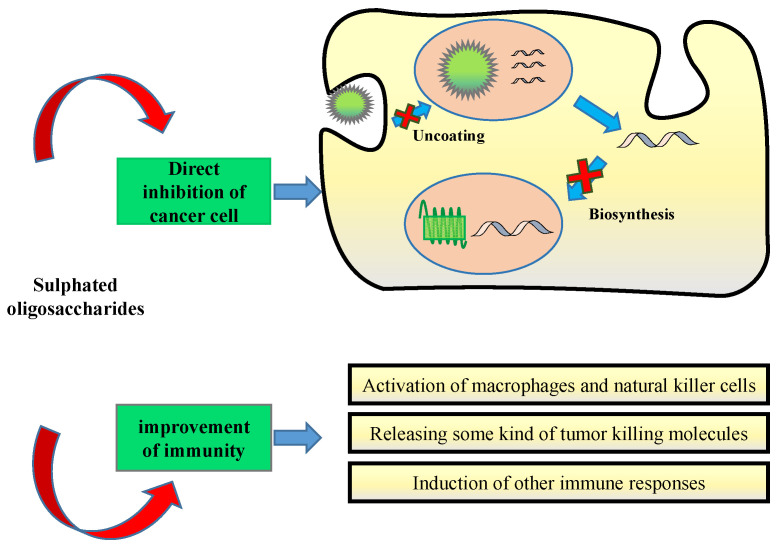
Anticancer activities of FOSs through two common mechanisms: direct and indirect anticancer activities that affect both tumor cells and their surrounding microenvironment.

Recently, anticancer activities of FOSs have been demonstrated through two common mechanisms: direct and indirect anticancer activities that affect both tumour cells and their surrounding microenvironment (Figure 4). In the former, FOSs directly inhibit cancer cells or induce tumour-cell apoptosis. In the latter, FOSs stimulate the host immune defence system (including activating macrophages, lymphocytes, and natural killer cells) and regulate the gut microbiota.

Apoptosis is a form of programmed cell death that plays a critical role in eliminating unnecessary or unhealthy cells. Numerous studies have found that sulphation present on carbohydrate moieties shows anti-proliferative activity in tumour cells by causing a cell cycle arrest and apoptosis [59]. FOSs with DP 2–6, with approximately five sulphate groups per sugar, showed significantly inhibited growth of the human malignant melanoma cell line SK-MEL-28, and better activity than fucoidan [60]. Another report on LWMF also showed higher inhibitory activity against breast cancer cells (MCF-7), human stomach cancer cells (AGS), and human liver cancer cells (HepG-2) than fucoidan [61]. A unique low-MW FOSs obtained from New Zealand *U*. *pinnatifida* inhibited the growth of MCF-7 and MDA-MB-231 cell lines (breast cancer cells) in a concentration-dependent manner, and induced apoptotic cell death through a caspase-dependent pathway [62]. Similarly, FOSs (containing approximately 72% and MW < 500 Da) induced MDA-MB-231 cell apoptosis, which is related to the activation of caspases and mitochondrial dysfunction, while decreasing the level of the Bc1-2 family [63].

In contrast, another anticancer approach is to use FOSs to enhance host immune defence by enhancing macrophages and natural killer cell functions. Macrophages are phagocytic cells, which have been considered for cancer immunotherapy in recent years, as they play an important role in host immune defence systems by killing tumour cells via phagocytosis, and releasing tumour-killing molecules, such as nitric oxide (NO), tumour necrosis factor (TNF-α), reactive oxygen species (ROS), interleukin (IL)-1β, and IL-6 [64]. FOSs with a MW < 10 kDa were degraded from *U. pinnatifida* and effectively triggered NO release, enhanced iNOS levels, and induced TNF-α and IL-6 secretion in RAW264.7 macrophages. The results also demonstrated that the mechanism of macrophage expression is related to the MAPK and NF-KB signalling pathways, which are the major regulators of inflammation and apoptosis in cancer cells [42]. Hwang et al. reported that an FOSs with a MW < 3000 Da significantly activated the immune defence system by improving NK cell activity, phagocytic activity, lymphocyte proliferation, and cytokine production [65].

Furthermore, mainstream cancer treatment sometimes incorporates the complementary therapy approach, which is a broad set of non-mainstream practices for the use of natural products together with conventional medicine. A previous study indicated that FOSs may serve as an efficient complementary therapy for cancer. Huang et al. demonstrated in vitro that FOSs and fluoropyrimidine combination chemotherapy improved therapeutic efficiency by inhibiting cancer cells. FOSs enhanced the suppressive effects of 5-FU on cancer cell migration through the c-mesenchymal–epithelial transition/matrix metalloproteinase-2 signalling pathway in HCT116 and Caco-2 cells [66]. The application of FOSs as a complementary therapy for cancer treatment has been investigated in clinical studies. Tsai et al. reported that FOSs with an average MW of 800 Da and sulphate content of approximately 39% (*w*/*w*) were used as a supplemental therapy combined with chemo-target agents in patients with metastatic colorectal cancer. They found that FOSs are an auxiliary therapy for metastatic colorectal cancer and improve the rate of disease control [67].

In summary, FOSs showed direct and indirect anticancer activity and could be developed as an anticancer agent. It would be notable to study the interactions between FOSs and conventional chemotherapeutic agents in the treatment or management of cancer. Clinical practices can provide large amounts of data and more convincing evidence; therefore, we only investigated clinical practices and obtained data to fully understand the therapeutic effect of FOSs.

### 5.3. Prebiotic Activity

Prebiotics are well known as health-promoting functional foods and nutraceuticals. Prebiotic oligosaccharides are usually indigestible by the stomach and upper gastrointestinal tract, and when they reach the large intestine, they are fermented and utilised by the gut microbiota [68,69]. This process transmits numerous health benefits to humans by producing numerous functional metabolites and modulating the intestinal ecosystem.

Maintaining a balance in the intestinal ecosystem is important to host health, as gut microbiota dysbiosis may increase the risk of some diseases, such as inflammatory bowel diseases, central nervous system disorders, cardiovascular diseases, and many kinds of cancers [70,71]. Several studies have reported that prebiotic oligosaccharides effectively maintain a favourable balance in the gut microbiota. Currently, fructo-oligosaccharides, galacto-oligosaccharides, xylo-oligosaccharides, and isomalto-oligosaccharides with prebiotic properties are commercially available [72]. Although FOSs are neither commercially available nor approved for use in functional food production, their role in the improvement of the gut microbiota is gaining increasing attention.

The ability of the gut microbiota to utilise poly/oligosaccharides is dictated by the repertoire of carbohydrate-active enzymes (CAZymes) encoded by their genomes, which normally include GH, polysaccharide lyases, and carbohydrate esterases (CE) [73]. The CAZyme-encoding genes act on fucoidan, and associated with the degradation of FOSs, belong to the families of CE4, GH29, GH107, S1_17 and S1_25 [27,28]. The diversity of CAZymes in the gut microbiota is linked to the complexity of the dietary fibre. Therefore, the dominant bacterial population in the gut is related to the oligosaccharides that are consumed by the host. In our previous study of in vitro fermentation of FOSs using a mixed culture of human faecal bacteria, the FOSs, prepared from *S*. *japonica* polysaccharides by mild acid hydrolysis, modulated the gut microbiota composition to increase the relative abundance of the phylum Bacteroidetes, and decrease the phylum Proteobacteria. Moreover, the production of SCFAs increased [74]. Such evidence was also found in vivo. The FOSs obtained from two species of sea cucumber, *Pearsonothuria graeffei* and *I*. *badionotus*, alleviated disordered intestinal bacteria in high-fat-diet-induced mice, especially by increasing the relative abundance of Bacteroidetes and decreasing of Firmicutes [75].

Bacteroidetes are one of the most abundant bacterial phyla in the human gut, and are considered primary utilisers of polysaccharides because they contains CAZyme-encoding genes that are necessary for the hydrolysis of poly-/oligosaccharide linkages [76]. Some species, such as *Bacteroides thetaiotaomicron* and *B*. *ovatus*, act as generalists able to cleave a wide range of glycosidic linkages of oligosaccharides, whereas others prefer to utilise specific glycosidic bonds or mono-/oligosaccharides which are released from poly-/oligosaccharides [77]. Bacteroidetes not only degrade the large MW of carbohydrates into smaller sugars, but also produce metabolites that may influence the formation of complex ecological networks and the diversity of the gut microbiota in the large intestine. The gut microbiotas exchange their metabolites with other bacteria, and these cross-feeding interactions play critical roles in the formation of diverse complex microbiota. Beneficial bacteria, such as *Lactobacillus* and *Bifidobacterium* species, are responsible for the cross-feeding behaviour of Bacteroidetes on oligosaccharides, because beneficial bacteria can utilise the smaller carbohydrates and metabolites produced by Bacteroidetes [78,79]. Some evidence has been obtained from previous studies on xylan-type polysaccharides. Co-fermentation of *Bifidobacterium animalis* with *Bacteroides* species, using xylan as a carbohydrate source, can enhance the growth of *B. animalis*. The results demonstrate that the prebiotic activity of xylans may be related to the cross-feeding of *Bacteroides* species, degrading xylan into smaller oligosaccharides and producing metabolites that are favourable to the *Bifidobacterium* species for growth [80]. Furthermore, the cross-feeding *B*. *ovatus* is also present on galactomannas, which share degraded products and metabolites together with *Bifidobacterium adolescentis* and *Lactiplantibacillus plantarum*, promoting their growth and increasing their production of SCFA [81]. FOSs prepared from *S*. *hemiphyllum* fucoidan showed a better prebiotic effect than their fucoidan, which promoted the growth of *Bifidobacterium lactis* [82]. Therefore, we believe that FOSs can support other beneficial bacterial growth and the diversity of the microbial community through cross-feeding interactions.

Prebiotic-derived functional metabolites, mainly SCFAs, exist and host a wide range of pharmacological effects, such as maintaining gut integrity, regulating glucose homeostasis, altering lipid metabolism, modulating the immune system, and protecting the cardiovascular system [83,84]. The major products of SCFAs include acetate, propionate, and butyrate, which are generated in the large intestine by beneficial bacterial fermentation of FOSs. SCFAs address their functions by activating specific G protein-coupled receptors, GPR43 and GPR41, which are also known as free fatty acid receptors 2 (FFR2) and 3 (FFR3), respectively, and are present in a variety of tissues [85]. Acetate normally stimulates the FFR2 at the cell surface, whereas butyrate binds and activates FFR3 [86]. Deng et al. reported that LWMF fraction LF2 ameliorated gut microbiota dysbiosis induced by a high-fat diet, by increasing the relative abundance of *Akkermansia muciniphila* and the production of SCFAs [87]. Recently, *A. muciniphila*, regarded as a probiotic, has been shown to degrade mucins or oligosaccharides as energy sources to produce SCFAs. The relative abundances of *A. muciniphila* and its derived SCFA are closely associated with the thickness of the colonic mucus layers and mucosal barrier integrity [88]. Acetate and propionate, the metabolites of oligosaccharide degradation by *A. muciniphila*, specifically stimulate GPR43, leading to modulation during inflammation and protection in high-fat-diet-induced mice [89].

In short, FOSs have potential prebiotic ability because they are indigestible in the upper gastrointestinal tract, possesse cross-feeding interaction with the gut microbiota and produce functional metabolites [15,90]. However, further studies are needed to determine the mechanisms by which specific gut bacteria utilise FOSs, and the isolation and purification of bacterial strains are necessary. Meanwhile, to investigate how specific microbiotas share their metabolites with other beneficial bacteria, metabolomics studies need to be conducted.

### 5.4. Antiviral Activity

Viral infections are a global public health problem that threaten human life and health. Although several vaccines have been explored for protection against specific viruses, they do not provide permanent protection. Moreover, some clinically available antiretrovirals have serious side effects, such as mitochondrial toxicity, hypersensitivity, and lipodystrophy [91]. However, effective and low-toxicity antiviral components have been identified from natural sources. Several polysaccharides and oligosaccharides derived from natural resources have shown excellent antiviral activity against several viruses [92].

FOSs prepared from *L*. *japonica* with a MW of 3700–7600 Da presented good anti-influenza viral activities both in vitro and in vivo. FOSs showed inhibitory effects on I-type influenza virus, adenovirus, and parainfluenza virus infection in Hep-2, HeLa, and MDCK cell lines, respectively. In vivo, intragastric administration of FOSs (5–10 mg/kg dosage) increased the survival time of mice exposed to the influenza virus, and also increased the organ indices of the thymus, spleen, and lung [93]. The antivirus mechanism of sulphated FOSs may associate with their ability in interfering the virus life cycle, including transcription, replication, and invasion [94].

*Hantaan orthohantavirus* is a global public health threat and may cause haemorrhagic fever with cardiopulmonary syndrome after infection. The mixture of FOSs fractions obtained from *F*. *evanescens* fucoidan, degraded by fucanases (GH107 family), showed anti-orthohantaviral activity. This FOSs mixture contains oligosaccharides with DPs ranging from 4–16, and with a high concentration of fuco-tetrasaccharide, comprising the repeating units of →3)-α-L-Fuc*p*2S-(1→4)-α-L-Fuc*p*2S(1→ [95]. The anti-orthohantaviral mechanism of FOSs was observed by molecular docking, which showed that 2*O*-sulphated fuco-tetrasaccharide is able to bind with AMRV and block the viral glycoproteins Gn/Gc and integrin β3 binding site of the host cells, to prevent synthesis of new viral proteins [95].

Currently, COVID-19, caused by severe acute respiratory syndrome coronavirus 2 (SARS-CoV-2), has a high mortality rate and has spread worldwide [96]. Patients infected with SARS-CoV-2 can develop pneumonia, severe symptoms of acute respiratory distress syndrome, neurological disorders, and multiple organ complications [97]. Emerging evidence suggests that some highly sulphated polysaccharides, such as heparin, heparan sulphates, glycosaminoglycans, and fucoidan, have potential antiviral activity against COVID-19 [98].

**Figure 5:**
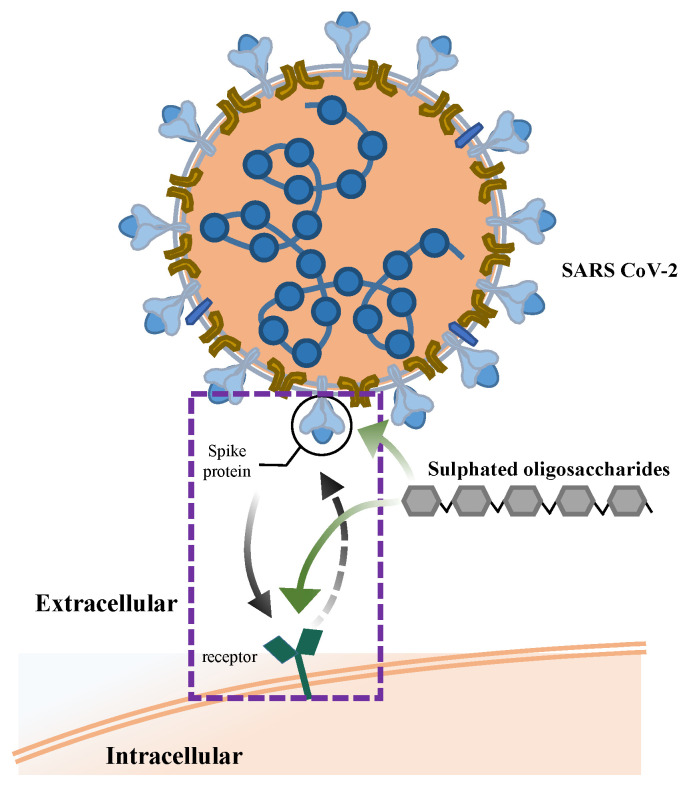
Schematic representation of the FOSs binding to the COVID-19 spike protein. FOSs (green arrow) suppress the viral attachment to receptor and subsequently its entry inside the host cell (black arrow).

Understanding the structural activity relationship and related mechanisms of the antiviral activities of sulphated polysaccharides against COVID-19 remains challenging and these are not yet fully understood. However, the key potential features can be identified from previous reports. First and foremost, it has been accepted by researchers that fucoidan and its degradation products, FOSs, contain negatively charged sulphate components, which can interfere with electrostatic interactions between the charged ions of the COVID-19 virus spike glycoprotein. Kwon et al. demonstrated that a specific sulphated polysaccharide binds tightly to the spike protein of COVID-19 in in vitro Vero cells infected experimentally, and demonstrated antiviral properties in host tissues by interfering with spike proteins binding to the heparin sulphate co-receptor to inhibit COVID-19 infection [98]. A similar finding was reported by Salih et al.: marine algae sulphated polysaccharides bind with an affinity for the receptor-binding domain of the COVID-19 spike protein and human angiotensin-converting enzyme-2, preventing the virus from affecting host cells [99] (Figure 5). LWMF derivatives, which were mainly composed of repeating units of α(1,4)-linked L-fucopyranosyl with different sulphation patterns, were synthesised by Koike et al. They found that compound 10 showed inhibitory activities against the interaction of heparin with a couple of mutant COVID-19 spike proteins, while no inhibitory activity was observed against factor Xa, which is a key serine protease of the coagulation cascade, that means without intrinsic anticoagulant [100].

Second, FOSs are known to modulate the immune system activity and may be capable of reducing COVID-19 infection. Several studies have shown that FOSs can stimulate the immune defence cells, such as macrophages, dendritic cell, T cells, and B cells, and the production of pro-inflammatory cytokines; these processes are able to induce the cytolytic killing of virus-infected cells [101].

Finally, FOSs are good prebiotics for modulating the gut microbiota and increasing the production of short-chain fatty acids (SCFAs), which are beneficial for preventing or reducing COVID-19 infection. Gut dysbiosis has been associated with infection by various viruses, and emerging evidence includes COVID-19; and the gut microbiota in COVID-19 patients shows an increasing abundance of opportunistic pathogens and decreasing abundance of beneficial bacteria [102]. Gut-microbiota-derived functional metabolites, such as SCFAs, play an important role in maintaining a good intestinal barrier and related immune functions, so they could potentially alleviate the disease in COVID-19 patients [103]. The current findings propose that the functions of FOSs in the intestinal barrier and modulation of the gut microbiota may potentially contribute to reducing the risk of COVID-19 infection and relieving the severity of COVID-19 symptoms.

The FOSs derived from fucoidan, which is derived from brown algae, shows low cytotoxicity and promising antiviral activities, although most results obtained are from in vitro experiments and still need more testing in vivo in future. We suggest that FOSs may have promising clinical applications in future.

## 6. Concluding Remarks and Prospects

Oligosaccharides have gained wide attention owing to their prebiotic properties and potential beneficial health implications. FOSs are derived from marine resources, which are abundant and renewable, have promise for many biological activities and are useful for treating various diseases. It is necessary to review the research into FOSs so far, to point out the potential for their application and exploitation. For example, FOSs can be potentially used in food ingredients or food preservatives due to their antioxidant activities. The use of FOSs in food preservatives preserves the quality of food and enhances the shelf life. In addition, FOSs can be potential applications in biomedical fields, such as drug carriers for efficient chemotherapy and anticancer agents.

Several reports have concentrated on the production of FOSs, and the enzymatic degradation of fucoidan into FOSs is a promising method; however, it has only been investigated in the laboratory. Further research should focus on the design and development of novel preparation methods that are suitable for FOSs in industrial scale-up processes. In addition, because FOSs can vary in their glycosidic linkages, MW, and sulphate content, pharmaceutical activities may also vary. Therefore, research on the structure–function relationship of FOSs will become a main focus for the application of FOSs in various areas. This review and the data summarized and discussed here can encourage innovative research on FOSs, and future research should focus on the preparation, biological activities, and regulatory mechanisms of FOSs, thus providing more fundamental data for their future application.

## Figures and Tables

**Table 1:** Sources of fucoidan derived from brown algae, sea cucumber, and sea urchin species.

Sources	Species
Brown algae	*Adenocystis utricularis*, *Ascophyllum nodosum* [5], *Bifurcaria bifurcate* [6], *Cladosiphon okamuranus* [6], *Coccophora langsdorfii* [7], *Cystoseira sedoides* [7], *Desmarestia intermedia* [6], *Dictyosiphon foeniculaceus* [6], *Dictyota dichotoma* [6], *Ecklonia kurome* [6], *Eisenia bicyclis* [6], *Fucus vesiculosus* [5], *F. serratus* [6], *F. spiralis* [6], *Laminaria digitate* [5], *L. saccharina* [5], *Saundersella simplex* [6], *Sargassum siliquosum* [6], *Scytosiphon lomentaria* [6], *Undaria pinnatifida* [6]
Sea cucumber	*Acaudina leucoprocta* [2], *A. molpadioides* [2], *Holothuria fuscopunctata* [2], *H. polii* [2], *Isostichopus badionotus* [2], *Ludwigothurea grisea* [2], *Stichopus horrens* [2], *Thelenota ananas* [2]
Sea urchin	*Arbacia lixula* [6], *Lytechinus variegatus* [6], *Strongylocentrotus droebachiensis* [8], *S. franciscanus* [6], *S. intermedius* [6], *S. pallidus* [8], *S. purpuratus* [6]

## Data Availability

No new data were created or analyzed in this study. Data sharing is not applicable to this article.
